# FACS-based purification of *Arabidopsis* microspores, sperm cells and vegetative nuclei

**DOI:** 10.1186/1746-4811-8-44

**Published:** 2012-10-17

**Authors:** Filipe Borges, Rui Gardner, Telma Lopes, Joseph P Calarco, Leonor C Boavida, R Keith Slotkin, Robert A Martienssen, Jörg D Becker

**Affiliations:** 1 Instituto Gulbenkian de Ciência, Rua da Quinta Grande 6, 2780-156, Oeiras, Portugal; 2 Cold Spring Harbor Laboratory, 1 Bungtown Road, 11724, Cold Spring Harbor, NY, USA; 3 Department of Molecular Genetics, The Ohio State University, 43210, Columbus, OH, USA

**Keywords:** Pollen, Microspore, Sperm cell, Vegetative nucleus, Plant germline, *Arabidopsis*, Fluorescence-activated cell sorting

## Abstract

**Background:**

The male germline in flowering plants differentiates by asymmetric division of haploid uninucleated microspores, giving rise to a vegetative cell enclosing a smaller generative cell, which eventually undergoes a second mitosis to originate two sperm cells. The vegetative cell and the sperm cells activate distinct genetic and epigenetic mechanisms to control pollen tube growth and germ cell specification, respectively. Therefore, a comprehensive characterization of these processes relies on efficient methods to isolate each of the different cell types throughout male gametogenesis.

**Results:**

We developed stable transgenic *Arabidopsis* lines and reliable purification tools based on Fluorescence-Activated Cell Sorting (FACS) in order to isolate highly pure and viable fractions of each cell/nuclei type before and after pollen mitosis. In the case of mature pollen, this was accomplished by expressing GFP and RFP in the sperm and vegetative nuclei, respectively, resulting in 99% pure sorted populations. Microspores were also purified by FACS taking advantage of their characteristic small size and autofluorescent properties, and were confirmed to be 98% pure.

**Conclusions:**

We provide simple and efficient FACS-based purification protocols for *Arabidopsis* microspores, vegetative nuclei and sperm cells. This paves the way for subsequent molecular analysis such as transcriptomics, DNA methylation analysis and chromatin immunoprecipitation, in the developmental context of microgametogenesis in *Arabidopsis*.

## Background

Plant germ cells differentiate late in ontogeny within gametophytes. In the male gametophyte (pollen grain), this process requires post-meiotic microspores to undergo two subsequent mitotic divisions, giving rise to the male germ unit (MGU) that is composed of a vegetative cell nucleus (VN) and two sperm cells (SC). The vegetative cell arrests cell cycle progression upon pollen mitosis I (PM I), while the two sperm cells originated from pollen mitosis II (PM II) are specified into gametes [1]. The germ cell lineage is immersed in the pollen vegetative cell, being dependent on this companion cell for transportation towards the embryo sac. However, the gametes are known to have their own unique molecular repertoire [2,3]. Several studies reported different methodologies to isolate generative cells of *Lilium longiflorum* (lily) [4,5] and sperm cells of *Oryza sativa* (rice) [6,7], *Zea mays* (maize) [8], and more recently from *Nicotiana tabacum* (tobacco) [9] and the dimorphic sperm cells of *Plumbago zeylanica*[10]. In *Arabidopsis*, technical difficulties in obtaining sufficient amounts of pure biological material constituted a major problem for purification of sperm cells, especially because of their small size. However, Fluorescence Activated Cell Sorting (FACS) has been successfully used to purify *Arabidopsis thaliana* sperm cells [2,11].

Microgametogenesis in Arabidopsis has proven to be an excellent model to identify novel mechanisms controlling cell cycle transitions, cell fate specification and epigenetic reprogramming (reviewed in [12]). Such studies also highlighted the importance of analyzing the components of the male germ unit independently, as they activate different transcriptional machineries and establish distinct epigenetic states [2,11,13]. The VN may participate actively in controlling heritable epigenetic modifications in the germline, as it activates expression of transposable elements (TEs) and produces a specific class of small interfering RNAs (siRNAs) that accumulate in the gametes [13]. Therefore, in order to understand epigenetic reprogramming throughout pollen development a simple and powerful method to co-purify the two differentiated types of nuclei in mature pollen, as well as their precursor microspore, was needed.

Here we describe a fast and reliable method to isolate *Arabidopsis* SC, VN and microspores, based on further development of previously reported techniques to isolate mature pollen using high-speed cell sorting [2,14]. Our first study described a fluorescent marker line specifically labeling differentiated SC in mature pollen, which allowed their FACS-purification and genome-wide transcriptional profiling [2]. Even though this method allowed obtaining pure and viable sperm cell fractions, it was laborious, time consuming and inefficient, considering the amount of plants needed as starting material. In addition, the need for DRAQ5 or other DNA dyes may become problematic for certain down-stream applications such as chromatin IPs, as it is known to interfere with chromatin condensation and nucleosome positioning [15,16].

This method was improved significantly by using stronger fluorescent markers and more efficient methodologies for pollen disruption, resulting in larger amounts of highly pure material at very high rates. As such, it also allowed co-purifying the VN from the same cell population.

## Results

### Co-purification of sperm cell and vegetative nuclei

**Figure 1:**
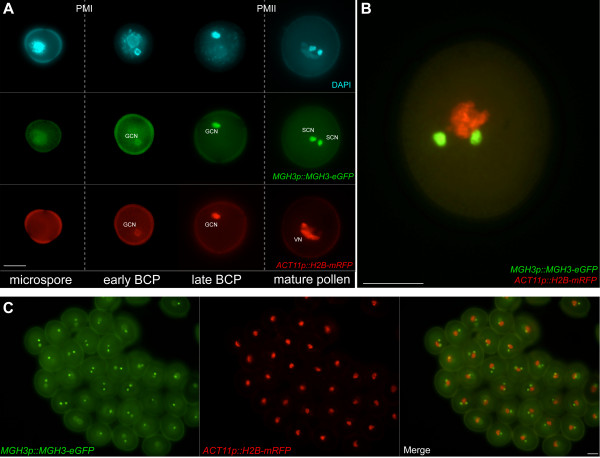
**Expression pattern of GFP- and RFP-fusion proteins during pollen development. (A) ***MGH3p::MGH3-eGFP* localizes in the generative cell nucleus (GCN) after the first pollen mitosis, and is strongly accumulated in the sperm cell nucleus (SCN) of mature pollen. *ACT11p::H2B-mRFP* is initially expressed in the GCN until late bicellular pollen (BCP). After the second pollen mitosis the expression of this transgene is down-regulated in the germline, and it becomes strongly expressed in the vegetative nucleus (VN). **(B)** Merged magnification of a mature pollen grain expressing both transgenes. **(C)** Population of mature pollen grains from double homozygous plants, confirming strong and stable expression of both transgenes. Scale bars: 10 μm.

In order to improve and simplify the SC-sorting method and to additionally co-purify the VN from the same genetic background, we generated a transgenic line expressing distinct fluorescent proteins in both nuclei. The *ACT11* promoter driving histone H2B fused to mRFP was used as a VN marker [17]. A homozygous plant harboring the *ACT11p::H2B-mRFP* transgene in Col-0 background was crossed with a sperm-specific marker line of the same ecotype, harboring a *MGH3p::MGH3-eGFP* construct, which encodes a male germline-specific histone variant [18,19]. *MGH3* is expressed as early as bicellular pollen, and is highly abundant in the sperm nuclei of mature pollen (Figure 1A, B). Progeny resulting from this cross were allowed to segregate by self-pollination, in order to obtain stable double homozygous plants (Figure 1C). Studying the expression pattern of both transgenes throughout pollen development revealed that *ACT11p::H2B-mRFP* is initially expressed in the generative cell (GC) of bicellular pollen, but preferentially expressed in the VN at the mature pollen stage (Figure 1A, B). We did not observe any pollen phenotypes in homozygous lines for both transgenes, and the plants are fully fertile, thus indicating that ectopic expression of an additional *H2B* gene in the VN and an extra copy of *MGH3* in the sperm cells did not result in any significant change in chromatin structure and dynamics in both nuclei.

**Figure 2:**
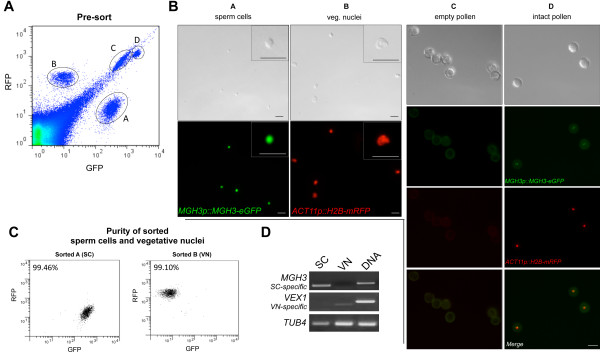
**Purification of sperm and vegetative nuclei by FACS. (A)** Four distinct cell populations are highlighted in the filtrate pre-sorting. Sperm cells (SC) nuclei are identified based on their strong GFP signal (Population A), whereas vegetative nuclei (VN) separate towards the opposite axis based on strong RFP signal (Population B). In addition the filtrate contains empty (Population C) and intact pollen grains (Population D). **(B)** Purity of sorted SC and VN fractions was confirmed by DIC and fluorescence microcopy; scale bar: 10 μm. Populations C and D were confirmed to represent empty and intact pollen, respectively; scale bar: 30 μm **(C)** Sorted SC and VN samples were stained with DAPI and run through a flow cytometer to check for purity. Purity is determined by measuring the percentage of SC and VN present within the total number of DAPI positive events, corresponding to all DNA-containing particles present in the sorted sample. **(D)** RT-PCR analyses confirmed that each fraction is enriched for cell-specific transcripts (*MGH3* for SC and *VEX1* for VN), and devoid of contaminating RNAs. *TUB4* was used as control.

Pollen grains were collected from open flowers by vortexing with Galbraith buffer [20] (see Materials and Methods), and released pollen was then disrupted by additional vortexing with glass beads. Enriched filtrates containing SC nuclei and VN released from broken pollen were co-purified by FACS based on their distinct fluorescence properties (Figure 2A). We inspected purity of sorted samples by microscopy (Figure 2B) and RT-PCR on SC and VC-specific transcripts. *MGH3* is only expressed in the SC and was not detected in the sorted VN fraction, while *VEX1*, a VC-expressed gene, was equally not detected in the SC fraction (Figure 2D), indicative of two pure populations. Re-analyzing sorted populations stained with 4',6-diamidino-2-phenylindole (DAPI) confirmed that the SC and VN populations were consistently more than 99% pure (Figure 2C). Furthermore, the ratio SC/VN before sorting was consistently 2:1, demonstrating a good recovery for both types of nuclei after pollen disruption (data not shown).

**Figure 3:**
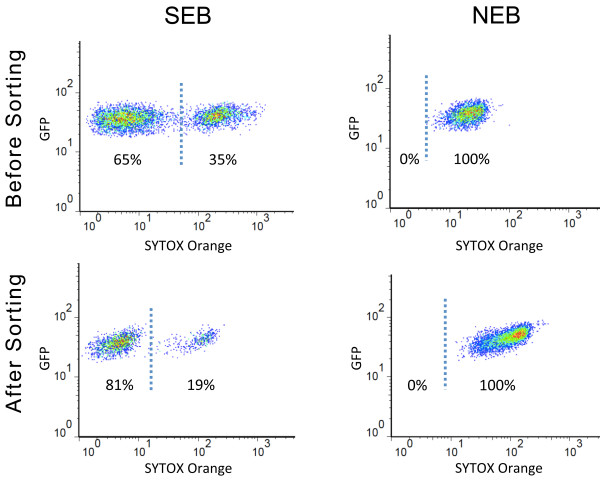
**Sperm cell viability before and after sorting.** Cell viability assays were performed by staining cells before and after FACS with SYTOX orange dye, which stains only dead cells. Sperm cells (SC) before and after sorting were confirmed to be viable only when prepared in Sperm Extraction Buffer (SEB), while in Galbraith’s buffer (NEB) we observed 100% staining.

As Galbraith’s buffer was originally conceived to sort and analyze nuclei by flow cytometry [20], we tested our FACS method using a sperm extraction buffer (SEB) that has been previously used to sort intact and viable sperm cells [2]. In order to test sperm cell viability before and after sorting, we stained the cells with SYTOX Orange dye that stains DNA of dead cells. As expected, sorted GFP positive events prepared in Galbraith buffer stained 100%, while only 19% of the cells stained after isolation in SEB (Figure 3). This result indicates that whenever intact and viable sperm cells are required for downstream applications, the cells should be prepared in SEB instead of Galbraith buffer. We could also confirm that this buffer is suitable for VN sorting, although the efficiency is significantly lower (data not shown). SYTOX staining can be additionally used to exclude dead sperm cells from sorted fraction.

The procedure was optimised to obtain higher yields of VN and SC nuclei/cells, maximising the number of sorted events per second, though the whole procedure can be scaled-down to approximately 150mg of fresh weight material in a 2 ml eppendorf tube. 0.8-1.5 million SC can be obtained from a 10 ml flower batch, while a 2 ml batch of open flowers yields approximately 100.000 sperm cells.

### Microspore purification by FACS

**Figure 4:**
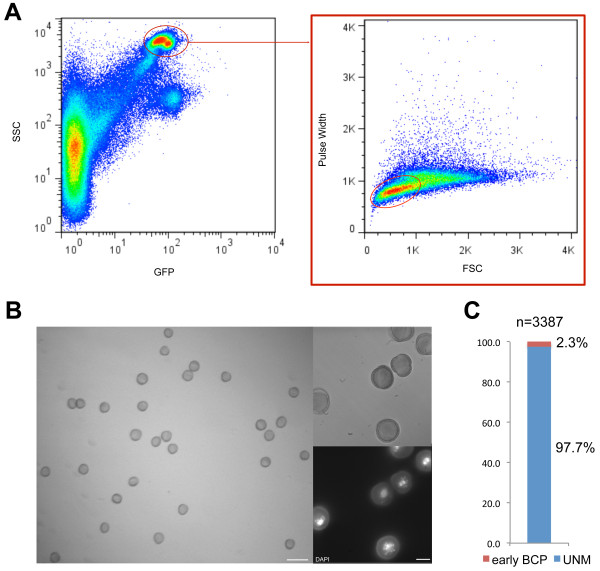
**Microspore sorting. (A)** Pollen population is characterized by an elevated high angle scatter (SSC) and autofluorescence (observed in the GFP channel using a 530/40 nm bandpass filter). Within this population, microspores (right panel, circular gate) can be differentiated from bicellular and tricellular pollen by their characteristic smaller size, captured by a diminished low angle scatter (FSC) and time-of-flight (Pulse Width), as compared to other stages of pollen development. **(B)** Sorted microspores were inspected by microscopy to show purity and integrity, as revealed by DAPI staining. Scale bars: 30 μm (left panel) and 10 μm (right panels). **(C)** Sorted microspores stained with DAPI were counted on a wide-field fluorescence microscope to confirm that most sorted cells are Uninucleate Microspores (UNM). BCP - Bicellular Pollen.

To understand the genetic and epigenetic mechanisms that control VN and SC differentiation, analysis of their precursor microspore cell is essential. Available methods to isolate *Arabidopsis* microspores relied on Percoll density gradients [21], and would not provide a sufficiently pure fraction suitable for genomic analysis at the DNA level, besides the problem of relatively low yield. Previous attempts to isolate microspores by flow cytometry were successfully applied in *Brassica napus*[22], however, to our knowledge these same methods were not tried in *Arabidopsis.* We used closed flower buds of wild type plants to collect all different stages of pollen development, by grinding in pollen extraction buffer (PEB) (see details in Materials and Methods). The method to purify SC and VN revealed that intact pollen grains have a high amount of autofluorescence (population D in Figure 2A). As such, we explored this property in addition to the smaller size of microspores in comparison with other stages of pollen development (Figure 4A). The sorted population was analyzed by microscopy and confirmed to contain mostly microspores (97.7%) (Figure 4B and C). A very small fraction (2.3%) of early bicellular pollen was also observed (Figure 4C). As these are probably cells that have just gone through the first pollen mitosis, they have identical size and autofluorescent properties as microspores, and are therefore impossible to distinguish with our FACS settings.

## Discussion

Gametogenesis and fertilization involve a series of genetic and epigenetic reprogramming events, often characterized by transcriptional activation of germline genes and repression of somatic gene expression, as well as dynamic changes of DNA methylation on a genome-wide scale. A comprehensive interpretation of such phenomena relies on analyzing isolated cell populations, and as such it demands methods for efficient and robust purification of each cell type involved in the process. We found that FACS coupled to nuclei-specific fluorescent markers provides fast and reliable results to isolate *Arabidopsis* sperm cell nuclei and vegetative nucleus fractions that are consistently more than 99% pure. Sorted nuclei are suitable for most molecular analysis such as DNA methylation profiling and chromatin immunoprecipitation. We have shown that our method allows the isolation of intact and viable sperm cells when prepared in SEB buffer, providing roughly 80% of viable cells in the sorted population. Moreover, as SYTOX dye stains DNA of dead cells, it can be used to sort only intact sperm cells.

Our results show that using fluorescent proteins under the control of strong cell-specific promoters eliminates the need for DNA dyes, e.g. SYBR Green as used previously to isolate VN and SC nuclei by flow cytometry [11,23]. The use of such dyes may enhance noise and interfere with downstream DNA analysis.

Besides the isolation of VN and SC, we developed a FACS-based protocol allowing the isolation of microspores from wild-type plants with unprecedented purity, accomplished again without the use of DNA dyes. These methods in combination with recent improvements in sample preparation, will allow fast and robust genome-wide analyses at the transcriptome and methylome levels from a very limited number of cells. As such, the genetic and epigenetic characterization of the *Arabidopsis* male gametophyte will provide the necessary data depth and resolution to boost our understanding of the key pathways involved in microgametogenesis in higher plants. Small RNA activity and epigenetic reprogramming of transposable elements in the germline [24] are particularly interesting for their defined role during post-fertilization processes such as genomic imprinting and heterosis.

## Methods

### Plant material, growth conditions and transgene cloning

Transgenic *Arabidopsis* plants were used for both sperm cell and pollen isolation. Plants were sown on soil and grown for 8 weeks in short-day conditions (8 h light at 21°C-23°C) and then transferred to long-day conditions (16 h light) to induce flowering. *MGH3p::MGH3-eGFP* was obtained by cloning 1.2 kb upstream of the MGH3 transcriptional start site, together with the MGH3 genomic sequence without the stop codon, into the pMDC107 vector [25] by gateway cloning. The *MGH3* coding sequence contains all endogenous introns, which might help for stable transcription and accumulation to high levels. Primers used for *MGH3p::MGH3-eGFP* cloning are presented in Additional file 1. Double homozygous plants harbouring *MGH3p::MGH3-eGFP* and *ACT11p::H2B-mRFP*[17] transgenes, were obtained by crossing individual homozygous lines. This seed stock is available from ABRC under stock number CS67829.

### Purification of sperm cells, vegetative nuclei and sperm nuclei

Open flowers were collected into a 50 ml falcon tube, filled roughly with a volume of 10 ml of fresh material. Flowers were vortexed at medium speed (Fisher Scientific, TOP-mix 3 IKA, speed 2000) in 10 ml of Galbraith buffer (45 mM MgCl_2_, 30 mM Sodium Citrate, 20 mM MOPS, 1% Triton-100, pH to 7.0) for 3 minutes, at room temperature. This crude fraction was then filtered through a Miracloth mesh (Calbiochem) to remove flower parts and centrifuged for 1 minute at 2600 g to pellet pollen. The supernatant was carefully removed with minimal disturbance and the pollen enriched pellet was resuspended in approximately 1.5 ml of fresh buffer. This pollen enriched fraction was then transferred to a 1.5 ml eppendorf tube containing 100 μl of acid-washed glass beads (425–600 μm, Sigma), and vortexed continuously at maximum speed (2500) for 4 minutes in order to break mature pollen grains. The fraction containing the released nuclei was then filtered through a 28μm mesh (SEFAR) to exclude unbroken and hydrated pollen. The nuclei enriched solution was ready for FACS at this point, which included debris from broken pollen. Hydrated pollen that remained intact and thus was retained in the filter can be recovered by washing the 28 μm mesh in new buffer and used in a second extraction step with the glass beads.

If intact and viable SC are desired for downstream applications (e.g. RNA extraction or *in vitro* manipulation) the whole procedure should be performed with sperm extraction buffer (SEB) (1.3 mM H_3_BO_3_, 3.6 mM CaCl_2_, 0.74 mM KH_2_PO_4_, 438 mM sucrose, 5.83 mM MgSO_4_, 7 mM MOPS at pH 6), which assures that 75-80% of the sorted sperm cells are viable and intact.

### Purification of Microspores from young flower buds

Closed flower buds (approximately 20 ml) were gently ground using mortar and pestle in 10 ml of pollen extraction buffer (PEB) (10 mM CaCl_2_, 2 mM MES, 1 mM KCl, 1% H_3_BO_3_, 10% Sucrose, pH 7.5) in order to release the spores. This crude fraction was initially filtered through Miracloth (Calbiochem) to remove bigger debris, and concentrated by centrifugation (800 g, 5 min) in 15 ml falcon tubes. The resulting yellowish pellet enriched in microspores was resuspended in 1.5 ml of PEB, and filtered through a 20 μm mesh (Partec, CellTrics) before FACS to further enrich for microspores and exclude tricellular and mature pollen.

### Fluorescence activated cell sorting

Fluorescent activated cell sorting was carried out with a MoFlo (Beckman Coulter, Fort Collins, USA) with a 488 nm laser (200 mW air-cooled Sapphire, Coherent) at 140 mW used for scatter measurements (Low angle or Forward Scatter, and High angle or Side Scatter; FSC and SSC, respectively) and for GFP excitation, and a 561 nm laser (50 mW DPSS, CrystaLaser) at 38 mW for RFP excitation. GFP and RFP were detected using a 530/40 nm and a 630/75 nm bandpass filters, respectively. FSC was used for triggering, and threshold had to be low to avoid missing the sperm cell population, whose size is on average 2.5 μm in diameter. Phosphate Buffer Saline (PBS) was used as sheath, and run at a constant pressure of 400 kPa (~60 psi). Frequency of drop formation was approximately 96,000 Hz. Even though pollen was present in the sample (broken and intact) it did not interfere with drop formation or break-off, as we were able to sort pollen under the same conditions as for SC and VN. Sorting rates were typically 2 million SC and 1 million VN per hour, i.e. an average rate of 500 SC and 250 VN per second, respectively.

Viability tests were performed with approximately 50.000 sorted SC and VN by staining with SYTOX Orange (Molecular Probes, Invitrogen) at a final concentration of 25nM. Sperm cells negative for SYTOX Orange and positive for GFP were considered viable (intact membrane) and considered compromised or to represent bare nuclei (only the nuclei stained), if positive for both SYTOX and GFP. SYTOX Orange was excited with the 488 nm laser and detected with a 580/20 nm bandpass filter in the Moflo.

Purity was determined by running aliquots of sorted cells in a CyAn ADP flow cytometer (Beckman Coulter, Fort Collins, USA). A 488 nm laser was used to excite both GFP and RFP, detected with 530/30 and 616/21 nm bandpass filters, respectively. 1 μM DAPI (Sigma) was added to the sorted cells and incubated on ice for 5 min, to discriminate between nuclei containing cells and electronic noise. DAPI was excited with a 405 nm laser and detected with a 450/40 nm bandpass filter.

Microspores were also purified by FACS using a Moflo high-speed cell sorter. The machine was used in a standard configuration, using a 100 μm ceramic nozzle with PBS running at a constant pressure of 200 kPa (~30 psi), and a drop-drive frequency of approximately 30,000 Hz. The 488 nm laser line was used for scatter measurements and autofluorescence excitation, which was detected in the GFP channel using a 530/40 nm bandpass filter. Microspores and other stages of pollen development were identified by their elevated high angle scatter (SSC) and autofluorescence properties (observed in the GFP channel). Within this population, microspores were selected by their characteristic smaller size, captured by a diminished low angle scatter (FSC) and time-of-flight (Pulse Width).

### RT-PCR

Semi-quantitative RT-PCR was performed with total RNA isolated from approximately 100,000 cells/nuclei that were sorted directly into Tri Reagent LS (Sigma). First-strand cDNA (Oligo-dT primed) was synthesized in 25 μl reactions using the MLV reverse transcriptase - RNaseH minus (Promega) according to manufacturer instructions. 2 μl of non-diluted cDNA was used as a template for 30 PCR cycles. Primers used are listed in Additional file 1.

## Competing interests

The authors declare that they have no competing interests.

## Authors' contributions

JDB initiated the project; FB, JDB, RG and RKS designed the experiments for isolation of sperm cells and vegetative nuclei; FB, JDB, RG, JPC and RM developed the strategy for isolation of microspores; FB, RG, TL, JPC, LCB and RKS carried out the experiments; FB, JDB and RG wrote the manuscript. All authors read and approved the final manuscript.

## Supplementary Material

Additional file 1 List of primers used in this study. Click here for file
