# Supplementary material for: FACS-based purification of Arabidopsis microspores, sperm cells and vegetative nuclei
Source: Plant Methods. 2012 Oct 17;8:44. doi: 10.1186/1746-4811-8-44 (PMC3502443; doi:10.1186/1746-4811-8-44)
Supplement: Additional file 1 — List of primers used in this study. [file 1746-4811-8-44-S1.pdf]

Additional file 1

Primers used in this study

Primer	Sequence (5' – 3')
VEX1_for	ATGGAAGATGAAATCGGTCTC
VEX1_rev	GCAGCATACATGATGACGTTG
MGH3_for	ACTAGACGACCGTACCGTGGT
MGH3_rev	CGATTCTATCTCACCCATCAA
TUB4_rt_for	ATCCCAAACAACGTCAAGTCC
TUB4_rt_rev	CTCTCCGGCTGTAGCATCTTGGTAC
Mgh3p_TOPO _F	caccTACTTCTCCGACCAAAAACTT
Mgh3 _TOPO_R	AGCACGTTCCCCACGAATGCGT
